# A pilot study of less invasive surfactant administration in very preterm infants in a Chinese tertiary center

**DOI:** 10.1186/s12887-015-0342-7

**Published:** 2015-03-14

**Authors:** Yingying Bao, Guolian Zhang, Mingyuan Wu, Lixin Ma, Jiajun Zhu

**Affiliations:** Department of Neonatology, Women’s Hospital, Zhejiang University School of Medicine, 1 Xueshi Road, Hangzhou, 310006 China

**Keywords:** Respiratory distress syndrome, Less invasive surfactant administration

## Abstract

**Background:**

Less invasive surfactant administration (LISA) to spontaneously breathing preterm infants has been reported to reduce the duration of mechanical ventilation and the incidence of bronchopulmonary dysplasia (BPD) in previous study. The objective of this study was to explore the feasibility and potential benefits of LISA in early preterm infants on nasal continuous positive airway pressure (nCPAP) compared to conventional endotracheal instillation.

**Methods:**

All infants with respiratory distress born at 28–32 weeks’ gestational age from January 2012 to December 2012 (n=90), who were eligible for exogenous pulmonary surfactant (PS) therapy were randomized to receive PS by intubation with an endotracheal tube (Intubation group, n=43), or by intubation using a catheter while on nCPAP (LISA group, n=47). Respiratory indices were recorded every 30 seconds during PS administration, and every 1 hour thereafter for the first day. The rate of mechanical ventilation (MV) in the first 72 hours, mean duration of both MV and nCPAP, mean duration of oxygen requirement and neonatal outcomes were recorded.

**Results:**

PS was successfully administered in 43 (100%) out of 43 babies using the conventional approach and in 46 (97%) out of 47 babies using LISA. The duration of both MV and nCPAP was significantly shorter in LISA group, when compared with intubation group. However, there were no significant differences in both the rate of MV in the first 72 hours and mean duration of oxygen requirement. There were also no differences in the mortality or in the incidence of bronchopulmonary dysplasia, intraventricular hemorrhage, retinopathy of prematurity and necrotizing enterocolitis, or in the duration of respiratory support.

**Conclusions:**

LISA in spontaneously breathing infants on nCPAP is an alternative therapy for PS delivery, avoiding intubation with an endotracheal tube. The method is feasible and potentially effective, and deserves further clinical trials.

**Trial registration:**

Current Controlled Trials ChiCTR-ICR-15006001. Registered 20 February 2015.

## Background

Large randomized controlled trials in very preterm babies show that initial nasal continuous positive airway pressure (nCPAP) therapy results in less mechanical ventilation (MV) and a trend towards lower risks for bronchopulmonary dysplasia (BPD) [1,2]. On the other hand, exogenous pulmonary surfactant (PS) therapy has been shown to substantially improve outcomes of preterm infants with respiratory distress syndrome [3,4]. Recognizing the merits of surfactant, especially when given early, some clinicians choose to intubate infants on CPAP solely for the purpose of giving PS, followed by immediate extubation. However, approximately 10% of those intubated solely for surfactant administration could not be extubated within 1 h [5]. In addition, intubation of the trachea with an endotracheal tube is an invasive procedure and may not be without risk.

Less invasive methods of delivering PS have been pursued. In 1992, Verder firstly described the use of a thin diameter catheter to apply surfactant to premature infants with emerging RDS on CPAP [6]. However, the observation study had not been paid much attention, until Kribs and colleagues used a similar method, which was called less invasive surfactant administration again in clinical observations (LISA), in Cologne [7]. The technique rapidly spread in Germany. Although no improved effects on long-term outcome have so far been demonstrated, these methods have shown promise in terms of achieving a clinical response without passing an endotracheal tube or using mechanical ventilation [8,9]. In previous studies, sedation and/or analgesia and/or atropine, to reduce secretions, may be given prior to the procedure. However, medication may interfere both with cardiovascular function (blood pressure) and respiratory effort.

The aim of our study was to assess the efficacy and the feasibility of LISA technique without medication and to compare the effects with the conventional management.

## Methods

### Population

The study was conducted in the Neonatal Intensive Care Units of the Women’s Hospital, Zhejiang University, School of Medicine from January 2012 to December 2012. The Ethics Committee of the Women’s Hospital, Zhejiang University, School of Medicine approved the study. A written informed consent for participation in the study was obtained from the parent of infants.

Inclusion criteria were: (1) infants born at 28 to 32 weeks gestational age. (2) Infants with RDS and need PS administration with 2 hr after birth. Exclusion criteria were: (1) infants who had been previously intubated and (2) infants with a congenital anomaly affecting respiratory function.

The diagnosis of respiratory distress syndrome (RDS) was based on the occurrence of classic signs of respiratory distress such as the need for oxygen, tachypnea, intercostal muscle retractions, grunting, and the exclusion of other causes of respiratory failure. The diagnosis was confirmed radiologically by reduced lung volumes, a reticulogranular pattern of lung consolidation, and air bronchograms [10]. Nasal continue positive airway pressure (nCPAP) was the initial means of respiratory support. Distending pressure ranged from 5 to 8 cm H_2_O, titrated according to oxygen requirement and work of breathing. Infants with signs of RDS, who were received nCPAP treatment and required nCPAP pressures ≧7 cm H_2_O and F_i_O_2_ ≧0.3 (28^+0^-29^+6^ weeks gestation) or ≧0.35 (30^+0^–32^+6^ weeks) to maintain SpO2 levels between 85% and 95%, were randomized to receive PS treatment (Curosurf, Chiesi Farmaceutici, Parma, Italy) at a dose of 200 mg/kg either by LISA procedure or conventional intubation. Infants were intubated, if F_i_O_2_ was ≧0.5, or if there was respiratory acidosis (pH <7.2) or significant apnea.

### Surfactant administration

During process of surfactant administration, the concentration of oxygen (F_i_O_2_) was adjusted using a blender to maintain oxygen saturation within the range of 85–95%.

#### LISA procedure

A 16 gauge, 130 mm vascular catheter (16G Angiocath, BD, Sandy, Utah, USA) was marked to indicate desired depth of insertion (28–29 weeks: 1.5 cm, 30–32 weeks: 2 cm). Direct laryngoscopy was performed, and the vascular catheter was inserted beyond the vocal cords to the required depth, and held in position at lips. If catheterization of the trachea was not possible within 20–30 s, the procedure was discontinued and attempted again once the baby was stable. Once the catheter was correctly positioned, surfactant was given at a standard dose as 5 boluses or more over 3-5 min. The tracheal catheter was immediately withdrawn. Infants were continued on nCPAP throughout the procedure. Positive pressure inflations were given by mask, if the infant developed apnoea or bradycardia.

#### Conventional intubation procedure

Surfactant instillation via endotracheal tube (ET) was performed with some brief mechanical ventilations, a standard dose of surfactant was always divided into 2 or 3 boluses. The endotracheal tube was withdrawn as soon as clinically possible after PS instillation, and the baby switched to nCPAP. The whole procedure took about 3 min and occurred without continuous distending pressure.

### Management after surfactant administration

After procedure, infants were stabilized on nCPAP. If FiO2 was >0.6, or if there was sustained respiratory acidos (pH <7.2) or repeated apnea, infants were intubated and receive MV. A further dose of surfactant (100 mg/kg) was given after intubation if clinically indicated. Care throughout hospitalization was as per routine for all infants, including monitoring for, and treatment of, patent ductus arteriosus (PDA), and screening for intraventricular haemorrhage (IVH) and retinopathy of prematurity (ROP) according to standard schedules of our center.

### Data collection

For each eligible infant, details during the PS instillation, including pulse oximetry saturation, heart rate and FiO_2_, were recorded prospectively every 30 seconds for about 3 min, along with pO_2_ and pCO_2_ values from blood gas samples before, and 1 h after PS administration. Changes in nCPAP pressure were recorded every 30 min in the first 4 hr, and at 12 and 24 h of life. Demographical data and early neonatal outcomes were recorded for all infants including need for intubation and mechanical ventilation in the first 72 h (and thereafter), further PS therapy. Mortality incidence of bronchopulmonary dysplasia (BPD) [11], PDA requiring medical and/or surgical therapy, intraventricular hemorrhage (IVH) grades III and IV [12], retinopathy of prematurity (ROP) greater than stage 2 [13] and necrotizing enterocolitis (NEC) on Bell stage II or III [14] were noted. The duration of respiratory support, including respiratory assistance (mechanical ventilation and/or nCPAP), oxygen therapy and intensive care admission were also recorded.

### Statistical analysis

We performed a power calculation analysis to determine the number of the study population. Since compared with the previous study [7,15], the gestational ages of infants were relatively older, hence to decrease the rate of intubation by 50%, about 60 infants in each group were needed to be recruited. Data were expressed as proportion, mean ± standard deviation (m ± SD) or median (interquartile range). Proportions were compared by Chi-square analysis. Continuous variables were compared by Student’s *t* test or Mann–Whitney *U* test according to their distribution. A p value <0.05 was considered statistically significant. Statistical analysis was carried out using the SPSS software, version 19.0 for Windows (SPSS, Chicago, IL, USA).

## Results

**Figure 1:**
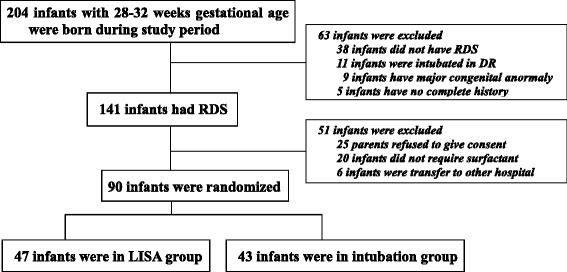
**Trial profile.**

During the study period, 204 infants with 28–32 gestational age, were born in our hospital. 141 infants were treated with nCPAP immediately after birth and assessed possibly for surfactant treatment; a total of 90 infants comprised the study group and were randomized into either LISA group or conventional intubation group, 47 infants in LISA group and 43 infants in conventional group were eligible for the statistical analysis (Figure 1). Demographic and clinical characteristics of the infants receiving surfactant by LISA were generally well matched with those managed by conventional intubation (Table 1).

**Table 1:** **Demographic and clinical characteristics**

	**LISA (n=47)**	**Intubation (n=43)**	**P value**
Gestation age (weeks), mean (SD)	29.1 (1.5)	29.3 (1.6)	0.54
Birth weight (grams), mean (SD)	1034 (221)	1087 (198)	0.24
Male gender, n (%)	28 (59.6)	26 (60.4)	0.93
Singleton, n (%)	21 (44.7)	19 (44.2)	0.96
Complete antenatal corticosteroids, n (%)	42 (89.4)	40 (93.0)	0.54
Cesarean section, n (%)	35 (74.5)	33 (76.7)	0.80
1 Min Apgar score	6.4 (0.8)	6.3 (1.2)	0.64
5 Min Apgar score	8.7 (0.6)	8.8 (0.7)	0.47
FiO2 prior to surfactant administration	0.43 (0.05)	0.43 (0.08)	1.0
PEEP prior to surfactant administration	6.5 (0.8)	6.4 (1.1)	0.62

The LISA technique and conventional surfactant administration were performed by 2 neonatal consultants and 3 neonatal fellows, with successful administration of surfactant in every case. A second or more catheterization attempts were required in 10.6% of infants who received PS by LISA, and a second or more intubation attempts were required in 14.0% of control infants (5 out of 47 vs. 6 out of 43, p=0.63).

**Figure 2:**
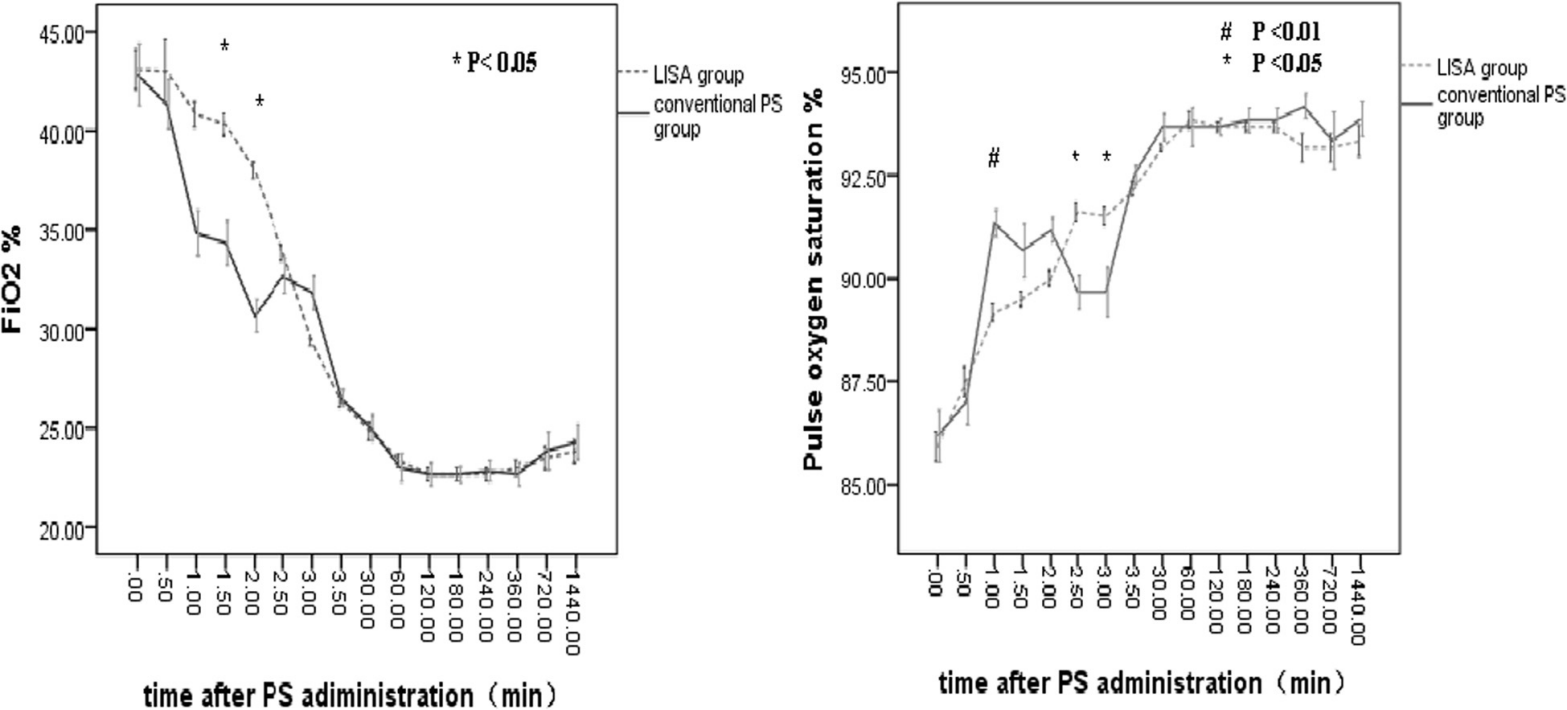
**Respiratory indices before and after surfactant administration-all infant, zero indicates the start of pulmonary surfactant administration.**

A decrease in PEEP and FiO_2_ was noted after LISA and conventional PS administration, sustained at least 6 hours. More fluctuations in heart rate and saturation were noted in conventional intubation group. During the whole procedure of PS administration, there was less fluctuation of FiO2 and oxygen saturation in infant receiving LISA. The final effects of providing PS using the two methods were similar (Figure 2). However, cases of surfactant reflux were more frequent in the LISA group (17 out of 47 vs. 5 out of 43, p=0.01).

Postnatal respiratory assistance outcomes (except the duration of both mechanical ventilation and nCPAP), morbidities (BPD, ROP, NEC and PDA), and mortality during the first 28 days of age were similar in the two groups (Table 2).

**Table 2:** **Postnatal respiratory management between LISA and control group**

	**LISA (n=47)**	**Intubation (n=43)**	**P value**
Time of first surfactant dose (min)	65 (20.7)	63 (25.3)	0.68
Two or more dose surfactant	8 (17.0)	5 (11.4)	0.44
pneumothorax	4 (8.9)	3 (7.0)	0.79
Supplemental O2 at age of 28 days	12 (25.5)	11 (25.6)	0.81
BPD	6 (12.8)	6 (14.0)	0.87
Mild	5 (10.6)	5 (11.6)	0.85
Moderate or severe, n (%)	1 (2.1)	1 (2.3)	0.51
PDA treat with medical therapy	28 (59.6)	26 (60.5)	0.93
PDA treat with surgery	1 (2.1)	0 (0)	0.34
IVH grades I or II	23 (48.9)	21 (47.7)	0.91
IVH grades III or IV	1 (2.1)	0 (0)	0.34
PVL	1 (2.1)	1 (2.3)	0.51
ROP > stage 2	0	0	ns
**Respiratory support**			
Mechanical ventilation during the first 72 hr, n (%)*	8 (17.0)	10 (23.3)	0.44
Mechanical ventilation days	6.7 (3.5)	7.4 (4.3)	0.40
nCPAP, n (%)^#^	47 (100)	43 (100)	ns
nCPAP days	10.9 (7.8)	12.3 (8.4)	0.41
Both MV and nCPAP days	13.2 (6.7)	15.9 (5.1)	0.03
Oxygen, n (%)*	38 (80.8)	34 (79.1)	0.83
Oxygen days	20.7 (5.2)	22.3 (6.9)	0.22
In hospital days	68.5 (17.3)	70.1 (22.5)	0.70
Died during the first 28 days	1 (2.1)	0 (0)	0.34

^#^need NCPAP during the hospitalization.

*need oxygen by mask or nasal tube during the hospitalization.

## Discussion

RDS is a common condition affecting very preterm infants, who often require surfactant replacement therapy. More and more preterm infants with respiratory distress are now treated with nCPAP soon after birth. The issue that intubation is solely required for the purpose of surfactant administration has risen. The operation of LISA aims at reducing the failure rate of nCPAP and minimizing intubation injury. However, LISA is not the “one size fits all” solution for surfactant therapy in premature infants [16]. The optimal gestational age and risk factors for failure of LISA are still not determined [17,18].

In this pilot randomized controlled trial, we show that LISA can be successfully applied to 28–32 gestational age infants with RDS who are receiving nCPAP, with no significant procedural complications. Moreover, the technique of LISA was relatively easy to learn: Given the initial lack of experience with LISA, there were a relatively low proportion of cases requiring a second catheterization attempts (2 out of the first 10 infants). We speculate that a narrow bore designed catheter, instead of conventional endotracheal tube was the main reason for the ease, because an external diameter about 1 mm of the catheter could be passed down and easily through the vocal cords. In our experience, the most difficulty of the method was how to perform direct laryngoscopy and expose the vocal cords on the infant with RDS on NCPAP. In the pilot trial, one baby was intolerant to LISA: the infant became and remained apneic while the catheter was intratracheal. This complication is rarely described in the literature. Two issues need to be discussed. One is the issue of sedation. The rate of sedation before the operation of LISA varied from about 0 in the study of Kanmaz, H. G et al.[19] to 26% in AMV study [15] or more in other study [20]. Although there was no consensus that sedation should be use before the operation of LISA, use of sedation may be a good choice. Further study of LISA may be focused on the issue of premedication. The other is the result of excessive individual sensitivity to airway manipulation, which was probably the cause of apnea in our infants. Thus, we infer that LISA may not be suitable for all premature infants.

In our study, we explored not only the respiratory indices pre-and post-surfactant administration, but also the change of FiO2 and SpO2 every 30 seconds during the procedure of surfactant administration by LISA, which were seldom reported in the previous papers [9,15,19-27]. We found an immediate and similar final physiological benefit, with a rapid and sustained reduction in FiO2 after surfactant administration by both LISA and conventional management. Although, it took longer (mean 3.2 min) to complete the whole LISA procedure, and more surfactant reflux was observed during LISA procedure.

Our results on the clinical benefits of LISA was slightly different from previous studies of minimally invasive PS instillation [19,23,24,27]. In the pilot study, we only found that LISA had benefit in reducing the duration of both ventilation and nCPAP, instead of reducing the rate of ventilation during the first 72 hours. Consistent with previous studies [15,20,23], we found that LISA had no benefit on reducing incidence of BPD. We speculate that this discrepancy could be explained by the difference in our patient demographics: infants who received PS in our study were an average of 29 weeks GA in our study. Our future studies will focus on the infants with less gestational age (24–28 weeks), who are more likely to receive mechanical ventilation and require oxygen for a longer time.

Fluctuations in oxygen saturation have been associated with certain neonatal diseases, including BPD, IVH and ROP [28-30]. We found conventional PS administration, especially with bag-mask ventilation, when compared with LISA was more likely to result in oxygen saturations greater than 95%, and infants would experience brief periods of desaturation after extubation (lasting about 3-5 min). We infer that we should pay attention to regulate tidal volumes and oxygen concentration and closely monitor the oxygen saturation during the whole procedure of PS administration.

Our study was an open-label, randomized, controlled pilot with a sample of about 90 infants. Since the optimal oxygen saturation for premature infants was changed to 90-95% in our NICU in 2014, we had to restrict our analysis to the data that had been gathered in 2013, with the pre-specified saturation ranges of 85-95%. This led to us not achieving the requisite sample size. Our results warrant further study. A large multicenter trial would be required to confirm that LISA reduces the duration of mechanical ventilation and oxygen therapy. Moreover, such studies would give us more information about the practicalities and complications of LISA.

## Conclusion

The LISA technique in spontaneously breathing infants on nCPAP is feasible, may prevent intubation with an endotracheal tube in certain premature babies. Further clinical trials are required to confirm the optimal strategy and the patient population in which the effect would be greatest.
